# Changes in Anterior and Posterior Corneal Elevation in Patients With Allergic Conjunctivitis

**DOI:** 10.3389/fmed.2021.788302

**Published:** 2021-11-24

**Authors:** Qian Wang, Fei Yu, Ziqing Feng, Weihua Li, Naiyang Li, Xinyue Du, Xuan Zhao, Saiqun Li, Jin Yuan

**Affiliations:** ^1^State Key Laboratory of Ophthalmology, Zhongshan Ophthalmic Center, Sun Yat-sen University, Guangdong Provincial Key Laboratory of Ophthalmology and Visual Science, Guangdong Provincial Clinical Research Center for Ocular Diseases, Guangzhou, China; ^2^Eye Center, Zhongshan City People's Hospital, Zhongshan, China

**Keywords:** allergic conjunctivitis, corneal tomography, corneal surface elevation, corneal posterior surface, corneal biomechanics

## Abstract

**Purpose:** To evaluate corneal elevation changes in patients with allergic conjunctivitis (AC) and to analyze their correlations with ocular allergy signs and corneal biomechanical alterations.

**Methods:** Thirty patients (30 eyes) with AC and twenty normal subjects (20 eyes) were included in this prospective study. All participants underwent a complete ocular examination, including corneal tomography by Pentacam and corneal biomechanics evaluation by Corvis ST. AC patients were evaluated for their eye rubbing frequency and ocular allergic signs.

**Results:** The elevation at the thinnest location (TE) and the central location (CE), the elevation difference at the thinnest location (TED) and the central location (CED), and the mean value of elevation difference in the central 4 mm zoom (MED) of both the anterior and posterior corneal surface were significantly higher in the AC group than in the normal group (*p* < 0.05 for all). In AC patients, only anterior corneal elevation parameters were positively correlated with eye rubbing frequency and ocular allergy sign severity (*p* < 0.05 for all), while the tomography and biomechanical index (TBI) was positively correlated with the elevation parameters of both the anterior and posterior corneal surfaces (*p* < 0.05 for all).

**Conclusion:** AC patients carry an increased risk of corneal ectasia. Posterior corneal elevation parameters are sensitive and reliable predictors of keratoconus (KC) risk in AC patients.

**Clinical Trial Registration:** https://clinicaltrials.gov/ct2/show/NCT04299399, identifier [NCT04299399].

## Introduction

Allergic conjunctivitis (AC) is a common ocular surface disease that affects more than 20% of the population (1). It is well established in the literature that AC is closely related to the occurrence of keratoconus (KC) (2–4). Various allergic ocular conditions, including vernal keratoconjunctivitis (VKC), atopic keratoconjunctivitis (AKC) and seasonal allergic conjunctivitis (SAC) or perennial allergic conjunctivitis (PAC), have been reported to increase the risk of developing KC (5). Although the underlying mechanisms remain unclear, increased inflammatory molecules and proteases and itch-provoked eye rubbing were thought to contribute to the development and progression of KC in AC patients (6).

AC has been found to be positively associated with early unset of KC. However, this association may still be underestimated in clinical analysis, since a considerable number of AC patients have subclinical presentation of KC (7). This subclinical population needs close monitoring for KC development. KC is characterized by progressive corneal thinning and a cone-shaped protrusion. It can be easily diagnosed in its intermediate to advanced stages, but an exact diagnosis of subclinical KC is still a major challenge because the diagnostic criteria remain to be defined (8). Several corneal topographic parameters, such as corneal elevation parameters and corneal thickness distribution indices, have been evaluated for their sensitivity to subclinical KC (9–14). Previous studies have shown that among the measured topographic parameters, including pachymetric parameters, elevation parameters and topometric parameters, anterior and posterior elevations had the greatest areas under the receiver operating characteristic curve (AUROCs) to discriminate subclinical KC from normal corneas (10, 13). KC was thought to start from the posterior surface of the cornea (15, 16). Moreover, taking into consideration that compared to the anterior corneal surface, the posterior cornea surface is less affected by corneal epithelial conditions and tear film stability, previous studies proposed posterior corneal elevations as more sensitive and reliable shape parameters for differentiating subclinical KC (15, 16).

Many studies have screened KC-like topographic and biomechanical changes in AC patients. KC-like tomography was observed in 11–20% of VKC patients (7, 17). Moreover, VKC was reported to cause a reduction in corneal biomechanics, as indicated by decreased corneal resistance factor (CRF) (18). Additionally, our previous study found alterations in the tomography and biomechanical index (TBI) in AC patients (19). In terms of posterior corneal surface changes in AC patients, studies have mainly concentrated on corneal aberrations and densitometry, and few have investigated posterior surface elevations. Barreto et al. (20) reported that VKC patients had significantly higher anterior and posterior elevations and pachymeter indices than healthy subjects. However, this study used Orbscan tomography IIz, which has been questioned by some researchers about its ability to accurately measure the posterior corneal surface. Because the Orbscan topographer works on the principle of slit scanning combined with Placido-disk technology, the posterior elevation map is derived mathematically from the Placido-disk reflection and 20 slit scans of the anterior segment (11, 14). In contrast, Pentacam uses a rotating Scheimpflug camera to capture 25 slit images and obtain a representation of the corneal shape, with which the posterior corneal elevations can be measured with high reproducibility and repeatability (21, 22).

In this study, we evaluated the corneal tomographic changes in AC patients using Pentacam, focusing on anterior and posterior surface elevation and pachymetric distribution. The correlations of these corneal tomographic changes with ocular allergic signs and corneal biomechanical changes were further analyzed. The purpose of this study was to elucidate the risk of KC development in AC patients and to identify sensitive indicators to screen high-risk patients for early intervention.

## Materials and Methods

### Participants

This prospective case-control study was conducted at the Zhongshan Ophthalmic Center, Sun Yat-Sen University and was approved by the Ethics Committee of the study hospital (2020KYPJ008). This trial has been registered at ClinicalTrials.gov as trial number NCT04299399. All subjects or responsible relatives signed informed consent forms before the study.

In this study, thirty AC patients and twenty normal subjects were included. The diagnosis of AC was determined according to the presence of typical clinical manifestations, including itching, redness, conjunctival hyperemia, palpebral conjunctival papillae or Horner-Trantas dots. Only patients with a history of AC for more than 2 years were enrolled. Age-matched subjects with no remarkable medical or ocular history except for refractive error served as normal controls. The exclusion criteria were: high refractive errors (spherical equivalent >6D and/or astigmatism >2D), active ocular inflammatory diseases other than AC, previous ocular surgery or disease, systemic diseases that might lead corneal abnormalities, soft contact lens wearing within 2 weeks and rigid contact lens wearing within 1 month.

### Examination Methods

Eye rubbing frequency and ocular allergy signs were evaluated in AC patients as reported in our previous study (19). Briefly, eye rubbing frequency was assessed on a scale from 1 (absent) to 5 (constant eye rubbing). Each objective ocular allergy sign (including conjunctival hyperemia, swelling, papillae, and corneal epithelial disorder) was graded on a scale from 0 (none) to 3 (severe) by the clinician.

All participants underwent a complete ophthalmic evaluation, including slit lamp examination, corneal tomography with Pentacam (Oculus, Inc., Wetzlar, Germany) and corneal biomechanics evaluation with Corneal Visualization Scheimpflug Technology (Corvis ST) (Oculus, Inc., Wetzlar, Germany). All measurements were performed by a single examiner to minimize interobserver variation.

**Figure 1:**
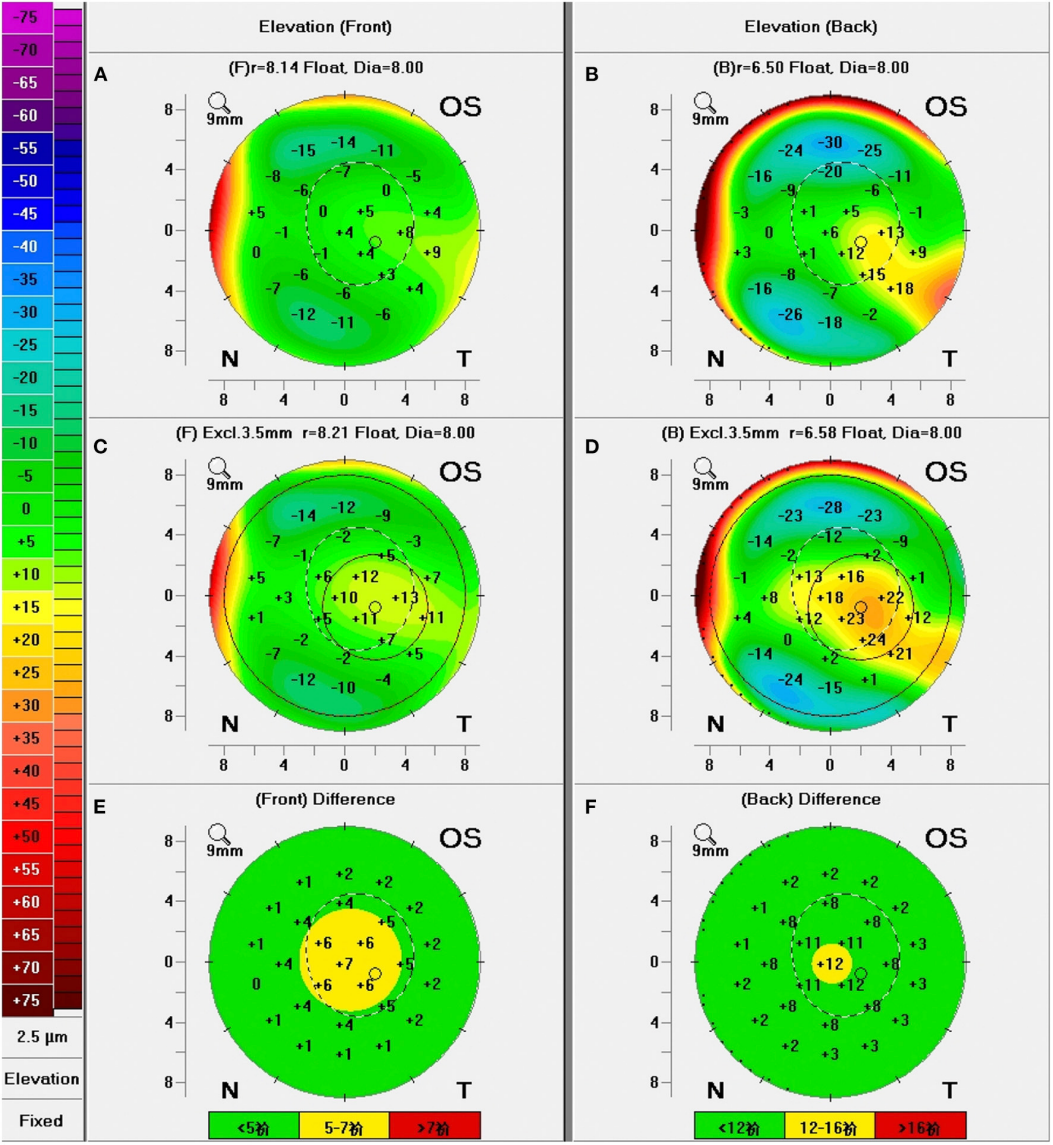
A representative example of elevation maps in patients with allergic conjunctivitis measured by Pentacam. **(A,B)** Anterior and posterior corneal elevations correlated to the standard best-fit sphere (BFS) reference surfaces; **(C,D)** Anterior and posterior corneal elevations correlated to the enhanced BFS reference surfaces (with exclusion of a 3.5-mm optical zone in the thinnest portion of the cornea); **(E,F)** Anterior and posterior corneal elevation differences between the elevation values correlated to the standard BFS and the enhanced BFS.

The following Pentacam parameters were analyzed: (1) topographic parameters of the anterior and posterior corneal surface, including keratometric values [flat keratometry (K1), steep keratometry (K2), mean keratometry (Kmean), maximum keratometry (Kmax)], astigmatism and Q value; (2) Pachymetric parameters, including central and thinnest corneal thickness (CCT and TCT), pachymetric progression indices [maximum (PImax), minimum (PImin) and average (PIavg)], and Ambrosio relational thickness indices [maximum (ARTmax) minimum (ARTmin) and average (ARTavg)]. On the Belin/Ambrósio enhanced ectasia display, pachymetric progression indices (PPIs) refer to the rate of corneal thickness changes along each meridian starting from the thinnest corneal point. The maximum, minimum and average PPI are recorded. Relational thickness indices express the ratio of the TCT to each of the above three PPIs, as expressed in the following formulas: ARTavg = TCT/PIavg; ARTmin = TCT/PImin; ARTmax = TCT/PImax (16); and (3) Anterior and posterior corneal elevations, including elevations at the thinnest and central locations (TE and CE), mean elevation in the 4 mm central zone (ME), elevation differences at the thinnest and central locations (TED and CED) and in the central 4 mm zone (MED). Corneal elevations were defined as the distances between the corneal surface and the best-fit sphere (BFS) reference surfaces set at 8-mm diameter. Corneal elevation difference values were taken as the differential changes in corneal elevation values between the BFS and the enhanced BFS (with exclusion of a 3.5-mm optical zone in the thinnest portion of the cornea) obtained with Belin/Ambrósio enhanced ectasia display software. An example of the data output for corneal elevations assessment is shown in Figure 1. The corneal biomechanical parameters measured by Corvis ST have been presented in detail in our previous article (19). TBI was selected for analysis in this study because we previously found that only TBI showed significant differences between AC patients and normal subjects.

### Statistical Analysis

To prevent bias, one eye of each participant was selected for analysis (for AC patients, the eye with more severe AC symptoms or signs was selected; for normal subjects, the eye was randomly selected). Statistical analyses were performed using SPSS software (version 25.0; SPSS, Inc., Chicago, Illinois, USA). The normality of the continuous variables was evaluated by the Kolmogorov–Smirnov test. Comparisons of continuous data between groups were performed by applying independent-sample *t*-tests. Categorical variables were compared using the χ^2^ test. Correlations between the continuous and ranked data were analyzed by Spearman's correlation test. *p* < 0.05 was considered statistically significant.

## Results

### Patient Characteristics

Twenty eyes of 20 normal subjects and thirty eyes of 30 AC patients were included in this prospective study. The demographics of the study population are shown in Table 1. There were no significant differences in age, sex, intraocular pressure (IOP) (measured by Corvis ST), spherical power (S), cylinder power (C) or spherical equivalent (SE) between the two groups (*p* > 0.05 for all).

**Table 1:** Demographics of the normal and AC groups.

**Characteristics**	**Normal**	**AC**	***p***
	**(*n* = 20)**	**(*n* = 30)**	
Age, mean ± SD	18.75 ± 7.99	19.37 ± 10.59	0.83
Sex, male, *n* (%)	13 (65.0%)	20 (66.7%)	0.90
IOP (mmHg)	16.03 ± 2.40	15.90 ± 1.96	0.84
S (D)	−1.92 ± 1.91	−0.79 ± 1.55	0.14
C (D)	−0.50 ± 0.45	−0.96 ± 1.29	0.18
SE (D)	−2.22 ± 2.07	−1.28 ± 2.65	0.24

*AC, allergic conjunctivitis; IOP, intraocular pressure, measured by Corvis ST; S, spherical power; C, cylinder power; SE, spherical equivalent; D, diopter; SD, standard deviation*.

### Corneal Topographic Parameters

Comparisons of the main corneal topographic parameters between the normal and AC groups are shown in Table 2. We found no significant differences in terms of the anterior corneal surface parameters between the two groups (*p* > 0.05 for all). The astigmatism and the Q value of the posterior corneal surface were significantly higher in the AC group than in the normal group (*p* = 0.02 and *p* = 0.01, respectively). The remaining topographic parameters of the posterior surface were not significantly different between the two groups (*p* > 0.05 for all).

**Table 2:** Comparison of corneal topographic parameters between the normal and AC groups.

**Parameter**	**Normal**	**AC**	***p***
	**Mean ± SD**	**Mean ± SD**	
**Anterior corneal surface**			
K1 (D)	42.41 ± 1.03	42.52 ± 1.28	0.75
K2 (D)	43.41 ± 1.05	43.87 ± 1.45	0.23
Kmean (D)	42.90 ± 1.01	43.20 ± 1.31	0.38
Kmax (D)	43.95 ± 1.13	44.46 ± 1.50	0.20
Astigmatism (D)	1.00 ± 0.54	1.34 ± 0.84	0.10
Q	−0.33 ± 0.11	−0.45 ± 0.26	0.05
**Posterior corneal surface**			
K1 (D)	−6.08 ± 0.16	−6.11 ± 0.21	0.59
K2 (D)	−6.40 ± 0.16	−6.5 ± 0.28	0.15
Kmean (D)	−6.23 ± 0.15	−6.29 ± 0.22	0.25
Astigmatism (D)	0.29 ± 0.12	0.38 ± 0.16	0.02^*^
Q	−0.26 ± 0.10	−0.34 ± 0.11	0.01^*^

*AC, allergic conjunctivitis; K1, keratometry of the flattest meridian; K2, keratometry of the steepest meridian; Kmean, mean central keratometry; Kmax, maximum keratometry; Q, Q value; D, diopter; SD, standard deviation*.

* *Statistically significant (p < 0.05)*.

### Corneal Pachymetric Parameters

Comparisons of the main corneal pachymetric parameters between the normal and AC groups are presented in Table 3. No significant difference was observed in any of the pachymetric parameters between the two groups (*p* > 0.05 for all).

**Table 3:** Comparison of corneal pachymetric parameters between the normal and AC groups.

**Parameter**	**Normal**	**AC**	***p***
	**Mean ± SD**	**Mean ± SD**	
CCT (μm)	550.15 ± 21.33	554.10 ± 24.59	0.56
TCT (μm)	547.35 ± 23.45	550.43 ± 25.72	0.75
PImin	0.73 ± 0.11	0.73 ± 0.11	0.82
PImax	1.28 ± 0.10	1.36 ± 0.19	0.09
PIavg	1.03 ± 0.09	1.05 ± 0.12	0.61
ARTmin	761.30 ± 124.35	776.30 ± 150.19	0.71
ARTmax	429.55 ± 45.79	415.97 ± 72.42	0.46
ARTavg	533.65 ± 58.67	533.07 ± 80.80	0.94

*AC, allergic conjunctivitis; CCT, central corneal thickness; TCT, thinnest corneal thickness; PI, pachymetric progression index; ART, Ambrosio relational thickness; min, minimum; max, maximum; avg, average; SD, standard deviation*.

### Corneal Elevation Parameters

Comparisons of the corneal elevation parameters of both the anterior and posterior corneal surfaces between the normal and AC groups are shown in Table 4. The TE, CE, TED, CED, and MED of both the anterior and posterior corneal surfaces were significantly higher in the AC group than in the normal group (*p* < 0.05 for all).

**Table 4:** Comparison of corneal elevation parameters between the normal and AC groups.

**Parameter**	**Normal**	**AC**	***p***
	**Mean ± SD**	**Mean ± SD**	
**Anterior corneal surface**			
TE (μm)	2.80 ± 1.24	4.07 ± 2.49	0.04^*^
CE (μm)	2.05 ± 0.94	3.33 ± 2.45	0.03^*^
ME (μm)	0.12 ± 0.31	0.18 ± 0.60	0.67
TED (μm)	3.40 ± 1.10	4.67 ± 2.22	0.02^*^
CED (μm)	3.50 ± 1.10	4.73 ± 2.23	0.03^*^
MED (μm)	2.60 ± 0.79	3.64 ± 1.69	0.01^*^
**Posterior corneal surface**			
TE (μm)	5.00 ± 2.70	7.73 ± 3.23	<0.01^*^
CE (μm)	0.35 ± 2.06	1.90 ± 2.64	0.03^*^
ME (μm)	0.56 ± 0.64	0.48 ± 0.75	0.69
TED (μm)	3.90 ± 2.36	6.13 ± 2.91	0.01^*^
CED (μm)	4.00 ± 2.70	6.57 ± 3.08	<0.01^*^
MED (μm)	2.99 ± 2.01	4.95 ± 2.29	<0.01^*^

*AC, allergic conjunctivitis; TE, elevation at the thinnest location; CE, elevation at the central location; ME, mean value of elevation in the central 4 mm zoom; ED, elevation difference; SD, standard deviation*.

* *Statistically significant (p < 0.05)*.

### Correlation of Corneal Elevation Parameters With Eye Rubbing Frequency and Ocular Allergy Sign Severity in AC Patients

The correlations of the corneal elevation parameters with eye rubbing frequency and ocular allergy sign severity in AC patients are presented in Table 5. The anterior corneal surface elevation parameters were positively related to eye rubbing frequency and the severity of ocular allergy signs, including conjunctival swelling, papillae and epithelial disorder (*p* < 0.05 for all). However, there was no correlation between the posterior corneal surface elevation parameters and eye rubbing frequency or ocular allergy sign severity (*p* > 0.05 for all).

**Table 5:** Correlation of altered corneal elevation parameters with eye rubbing frequency and ocular allergy sign severity in AC patients.

		**Anterior corneal surface**					**Posterior corneal surface**				
		**TE**	**CE**	**TED**	**CED**	**MED**	**TE**	**CE**	**TED**	**CED**	**MED**
Eye rubbing	r	0.251	0.414	0.424	0.452	0.462	0.277	0.023	0.247	0.226	0.211
	*p*	0.181	0.023^*^	0.020^*^	0.012^*^	0.010^*^	0.138	0.903	0.188	0.230	0.264
Hyperemia	r	0.011	0.082	0.099	0.109	0.077	−0.056	0.039	−0.221	−0.230	−0.256
	*p*	0.956	0.666	0.601	0.567	0.685	0.769	0.836	0.242	0.222	0.173
Swelling	r	0.257	0.312	0.403	0.420	0.409	0.075	0.024	−0.028	−0.075	−0.090
	*p*	0.170	0.093	0.027^*^	0.021^*^	0.025^*^	0.692	0.900	0.885	0.693	0.635
Papillae	r	0.280	0.318	0.455	0.455	0.452	0.103	0.141	0.008	−0.039	−0.024
	*p*	0.135	0.087	0.011^*^	0.011^*^	0.012^*^	0.588	0.458	0.967	0.838	0.899
Epithelial disorder	r	0.200	0.465	0.459	0.443	0.449	0.009	0.026	−0.035	−0.066	−0.070
	*p*	0.289	0.010^*^	0.011^*^	0.014^*^	0.013^*^	0.963	0.890	0.854	0.730	0.714

*AC, allergic conjunctivitis; TE, elevation at the thinnest location; CE, elevation at the central location; TED, elevation difference at the thinnest location; CED, elevation difference at the central location; MED, mean value of elevation difference in the central 4 mm zoom*.

* *Statistically significant (p < 0.05)*.

### Correlation of Corneal Elevation Parameters With the TBI in AC Patients

Table 6 shows the correlations of the corneal elevation parameters with TBI in AC patients. We found that TBI was positively correlated with TE (r = 0.608, *p* < 0.001), CE (r = 0.464, *p* = 0.010), CED (r = 0.727, *p* < 0.001), and MED (r = 0.750, *p* < 0.001) of the anterior corneal surface, as well as with TE (r = 0.563, *p* = 0.001) of the posterior corneal surface.

**Table 6:** Correlation of corneal elevation parameters with TBI in AC patients.

		**Anterior corneal surface**					**Posterior corneal surface**				
		**TE**	**CE**	**TED**	**CED**	**MED**	**TE**	**CE**	**TED**	**CED**	**MED**
TBI	r	0.608	0.464	0.754	0.727	0.750	0.563	0.156	0.341	0.318	0.323
	*p*	<0.001^*^	0.010^*^	<0.001^*^	<0.001^*^	<0.001^*^	0.001^*^	0.412	0.065	0.087	0.081

*AC, allergic conjunctivitis; TBI, tomography and biomechanical index; TE, elevation at the thinnest location; CE, elevation at the central location; CED, elevation difference at the central location; MED, mean value of elevation difference in the central 4 mm zone*.

* *Statistically significant (p < 0.05)*.

## Discussion

A close association between KC and AC has been well established in the previous literature (23–25). However, sensitive indicators for screening AC patients at high risk of developing KC are still under investigation. In our previous study, we showed that TBI could be used as an indicator of KC development risk in AC patients (19). In the current study, we found that both the anterior and posterior corneal elevation values were significantly higher in AC patients than in normal subjects and were further identified to positively correlate with the altered TBI in AC patients. Our findings provide additional evidence supporting that compared to normal subjects, AC patients have an increased risk of corneal ectasia, and corneal elevation could be used as a sensitive indicator of the risk of corneal ectasia in AC patients.

The typical corneal topography characteristics in KC patients include increased corneal curvature and corneal thinning. Barreto and colleagues (20) reported a significantly greater central curvature, thinner corneal thickness and higher pachymetric index in VKC patients than in normal subjects. However, contrary to the findings by Barreto et al., our study did not find significant differences in anterior and posterior corneal surface curvature parameters or pachymetric parameters between the normal and AC groups. There are controversial findings in different studies. Similar to our results, no significant change in K1, K2 or Kmean was found in VKC patients in Ekinci's research (26). The difference in the patient characteristics might be the reason for the different results of the two studies. Barreto's study included only active VKC patients, while our study also included patients with SAC and PAC. VKC, as a more severe form of AC, was reported to have higher levels of ocular inflammatory cytokines and active proteases than SAC and PAC (27, 28), which in turn could induce more severe ocular microstructure damage, indicated by a thinner corneal thickness and greater corneal curvature.

The corneal Q value is a parameter that reflects the corneal shape and represents the degree of corneal asphericity. A more negative Q value of the posterior corneal surface has been reported in early KC (15). In this study, we found greater astigmatism and a more negative Q value for the posterior corneal surface in the AC group than in the normal group. Our findings were consistent with what was reported by Dantas et al. (7). In their study, the Q value of the cornea was significantly more negative in the VKC group than in the normal group. A more negative Q value refers to a more prolate shape, with a steeper central cornea and flatter periphery. A previous study reported significant correlations between posterior elevation parameters and posterior Q values as well as posterior aberrations; hence, the authors speculated that the changes in posterior Q values and posterior corneal aberrations were attributed to alterations in corneal posterior elevation (15). Thus, we hypothesize that the more negative Q value and greater astigmatism of the corneal posterior surface in AC patients might also be induced by alterations in corneal posterior elevation.

Previous studies reported that corneal anterior and posterior elevation values were significantly higher in early KC patients than in normal subjects (10, 11, 13). Our study also found much higher corneal elevation values or elevation differences in either the anterior or posterior surface in AC patients than in normal subjects. However, whether KC-like changes in corneal elevation could predict KC development in AC patients still requires further investigation. Moreover, several studies have shown that posterior elevation parameters are more sensitive than anterior elevation parameters in differentiating KC from normal eyes (10, 12, 16). For example, Huseynli et al. (10) further showed that among the parameters measured by Pentacam, including corneal topographic parameters, pachymetric parameters, elevation parameters and topometric parameters, anterior and posterior elevation showed the highest AUROCs (0.935 and 0.897, respectively) to differentiate early KC patients from normal subjects. Therefore, among the parameters evaluated in their study, posterior corneal elevation changes were identified as the earliest sign of subclinical KC (15, 16). Additionally, in our study, we found that the increased elevations of both the anterior and posterior corneal surfaces were positively correlated with altered TBI, which was identified as a sensitive indicator of KC development risk in AC patients in our previous study (19). This result to a certain extent demonstrates the possibility and risk of KC occurrence in AC patients. In addition, our findings indicate that corneal elevation changes occurred earlier than the occurrence of abnormal corneal pachymetric distribution and increased corneal curvature in AC patients. Therefore, measurement of corneal elevation, especially of the posterior surface, may aid in evaluating the risk of KC development in AC patients. However, except for KC, higher corneal elevations have also been reported in other diseases such as patients with Familial Mediterranean fever (29). Even some normal subjects may have abnormal corneal elevations indicated by yellow color in the Belin/Ambrósio Enhanced Ectasia Display. Thus, it would be better to combine corneal tomography with other KC detection tools such as corneal biomechanical assessment and corneal epithelial thickness measurement to comprehensively evaluate the KC risk in AC patients.

Furthermore, we found that altered anterior corneal elevation was positively correlated with eye rubbing frequency and ocular allergy sign severity in AC patients. However, there was no significant correlation between posterior surface corneal elevation and ocular allergy sign severity. The anterior surface of the cornea could be affected by various factors. For example, AC patients often have unstable tear film and dry eye (30–33). Irregular tear films and the use of artificial tears in AC patients are factors influencing the corneal anterior surface (11, 26). Moreover, the corneal epithelial damage and thinning caused by excessive eye rubbing in AC patients could also induce increased irregularity of the anterior corneal surface. Therefore, anterior corneal elevation may not be suitable for use as an indicator of KC risk in AC patients due to its vulnerability to multiple factors, including ocular surface inflammation conditions, tear film stability and corneal epithelial defects.

Contrary to anterior corneal surface measurements, posterior corneal surface measurements were not influenced by the irregularity and allergic signs of the ocular surface. Although there was no significant correlation between altered posterior elevation and the severity of ocular allergy signs in AC patients, the alterations in posterior elevation were not necessarily independent of the inflammatory condition and the course of ocular allergy. Increased release of inflammatory cytokines, including matrix metalloproteinase (MMP)-1,−3,−7, and−13, interleukin (IL)-4,−5,−6, and−8 and tumor necrosis factor (TNF)-α and -β, induced by excessive eye rubbing in AC patients was reported to mediate pathological apoptosis of keratocytes and corneal fibroblasts, which could further induce alterations in the posterior corneal surface and corneal weakness (27, 34–36). Thus, posterior elevation parameters, which were mainly affected by corneal chronic and long-term inflammation, could be used as sensitive and stable indicators of KC risk in AC patients.

A limitation of this study is the small sample size; thus, studies with a larger sample size are needed to compare corneal tomography changes among different types of AC. Moreover, a follow-up study to explore corneal elevation changes over time is necessary.

In conclusion, compared to normal subjects, AC patients carry an increased risk of corneal ectasia, as indicated by higher corneal elevation parameters. Posterior corneal elevation parameters could be used as sensitive and stable indicators of KC risk in AC patients.

## Data Availability Statement

The raw data supporting the conclusions of this article will be made available by the authors, without undue reservation.

## Ethics Statement

The studies involving human participants were reviewed and approved by the Ethics Committee of Zhongshan Ophthalmic Center, Sun Yat-sen University (2020KYPJ008). Written informed consent to participate in this study was provided by the participants' legal guardian/next of kin.

## Author Contributions

JY and SL designed and supervised the study. QW and FY analyzed and interpreted the data and prepared the manuscript. ZF, WL, NL, XD, and XZ contributed to data collection. All authors contributed to the article and approved the submitted version.

## Funding

This work was supported by grants from the National Natural Science Foundation of China (82171015).

## Conflict of Interest

The authors declare that the research was conducted in the absence of any commercial or financial relationships that could be construed as a potential conflict of interest.

## Publisher's Note

All claims expressed in this article are solely those of the authors and do not necessarily represent those of their affiliated organizations, or those of the publisher, the editors and the reviewers. Any product that may be evaluated in this article, or claim that may be made by its manufacturer, is not guaranteed or endorsed by the publisher.
